# Case report: Illustrating associated malignancies in Paget’s disease using contrast-enhanced mammography

**DOI:** 10.3389/fonc.2024.1497506

**Published:** 2025-01-08

**Authors:** Luciano Mariano, Luca Nicosia, Anna Bozzini, Filippo Pesapane, Francesca Magnoni, Giovanni Mazzarol, Lorenza Meneghetti, Adriana Sorce, Enrico Cassano

**Affiliations:** ^1^ Breast Imaging Division, European Institute of Oncology (IEO), Istituto di Ricovero e Cura a Carattere Scientifico (IRCCS), Milan, Italy; ^2^ Department of Biotechnology and Life Sciences, University of Insubria, Varese, Italy; ^3^ Division of Breast Surgery, European Institute of Oncology, Istituto di Ricovero e Cura a Carattere Scientifico (IRCCS), Milan, Italy; ^4^ Division of Pathology, European Institute of Oncology (IEO), Istituto di Ricovero e Cura a Carattere Scientifico (IRCCS), Milan, Italy; ^5^ Postgraduation School in Radiodiagnostics, Università degli Studi di Milano, Milan, Italy

**Keywords:** contrast enhanced mammography, CEM, breast cancer, breast imaging, Paget carcinoma

## Abstract

**Introduction:**

The following presentation explores the diagnostic potential of Contrast-Enhanced Mammography (CEM) in evaluating and managing Paget’s Disease (PD) of the breast, particularly as an alternative or complementary tool to Magnetic Resonance Imaging (MRI) in cases where MRI is contraindicated or inconclusive.

**Clinical cases:**

Two clinical cases of PD diagnosed at our Breast Imaging Division between January and May 2024 were analyzed using CEM. These cases involved imaging techniques, including Digital Mammography (DM), Breast Ultrasound (US), MRI and CEM, alongside histopathological confirmation through nipple-areolar complex (NAC) punch biopsies. CEM identified disease extensions and NAC involvement that was not evident in conventional imaging in both cases. CEM findings influenced surgical decisions, leading to total mastectomies with reconstruction instead of conservative approaches. The cases highlighted CEM’s sensitivity and ability to delineate the disease extent comparable to MRI.

**Discussion and conclusions:**

PD often presents diagnostic challenges due to frequent associations with underlying malignancies that are undetectable by standard imaging. While MRI is the gold standard, its limitations, such as costs, contraindications, and false positives, warrant alternative methods. CEM demonstrated utility in diagnosing and staging PD, offering benefits in patient acceptability, cost, and sensitivity comparable to MRI. CEM is a promising diagnostic and planning tool for PD management, especially in MRI-infeasible cases. More extensive multicentric studies will be needed to validate CEM’s role in this context. CEM could enhance PD diagnostic workflows and treatment strategies, significantly impacting clinical outcomes.

## Introduction

Paget’s disease of the breast (PD) is a rare condition characterized by unilateral skin changes in the nipple-areolar complex (NAC) and often associated with underlying carcinoma (BC) (1). An accurate and timely approach, including clinical examination and imaging techniques integration, is crucial when PD is suspected, although a definitive diagnosis requires histological confirmation through a skin punch biopsy (1). PD patients frequently present with unilateral changes in NAC, such as itching, erythema, eczematous-erosion lesions, ulceration, nipple retraction, and serous or bloody discharge (2); however, obvious symptoms may not be shown, and diagnosis is made through pathological evaluation of the NAC during mastectomy (1–3). Conventional imaging techniques, like Digital Mammography (DM) and Breast Ultrasonography (US), can miss underlying malignancies in up to 65% of cases (4). Magnetic Breast Resonance Imaging (MRI) is an essential tool in detecting clinically occult cancer; it also plays a key role in preoperative planning, guiding decisions between conservative and demolition surgical treatments (4–6). However, MRI’s limitations, such as false-negative, costs and patient contraindications (e.g., claustrophobia or non-MRI-compatible devices), necessitate exploring alternative techniques (1, 7).

Contrast-enhanced Mammography (CEM) offers comparable performance to MRI in terms of sensitivity and has shown utility in PD evaluation due to its ability to highlight neo angiogenesis and greater patient acceptability (8). Despite limited studies, CEM holds promise as a complementary or alternative tool to MRI in the evaluation of PD. Here, we discuss two clinical cases to illustrate the potential of CEM in staging and managing PD.

## Clinical cases

**Figure 1:**
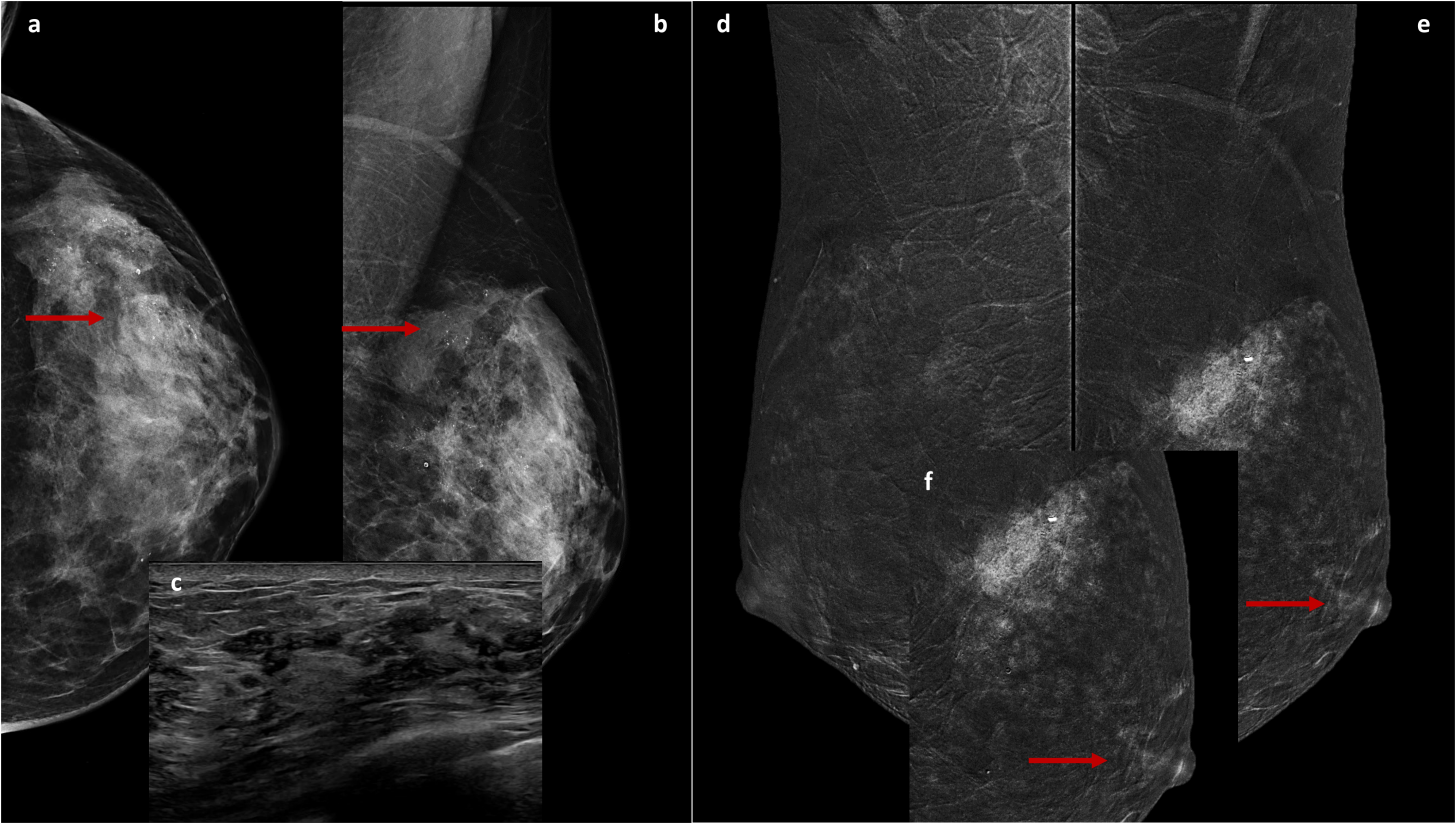
Cranio-caudal **(A)** and Medio-lateral-oblique **(B)** DM views of the left breast show suspicious fine and pleomorphic microcalcifications from the upper-outer quadrant to the outer equatorial region (arrows) corresponding to ectasia ductal structures with multiple contextual hyperechogenic spots on US **(C)**. The recombined medio-lateral-oblique CEM images **(D, E)** show, in addition to non-mass-like enhancement in the upper quadrants of the left breast (with a post-biopsy clip inside, **E**), a contrastographic impregnation of the ipsilateral NAC (arrows), highlighted in the focused magnification **(F)**.

The first case (**Figure 1**) involves a 46-year-old woman referred to our Breast Imagin Division for her annual follow-up examination. Her personal and family history of BC was negative, and a preliminary clinical assessment of the breasts revealed no alterations of NAC, palpable mass, or axillary adenopathy. Previous annual mammograms were unremarkable. DM revealed an area of fine pleomorphic microcalcifications in the left breast, with a segmental distribution extending from the upper-outer quadrant to the outer equatorial region, over approximately 6 cm, corresponding to ectasia ductal structures with multiple contextual echogenic spots on US. A US-guided biopsy diagnosed a high-grade *in situ* ductal carcinoma (DIN3). Subsequently, the patient underwent a staging CEM examination, refusing MRI due to claustrophobia. In addition to the known area, CEM showed contrast-enhancement of the ipsilateral NAC. A skin punch biopsy confirmed the PD diagnosis. Therefore, the patient was referred for left total mastectomy with reconstruction rather than a nipple-sparing procedure.

**Figure 2:**
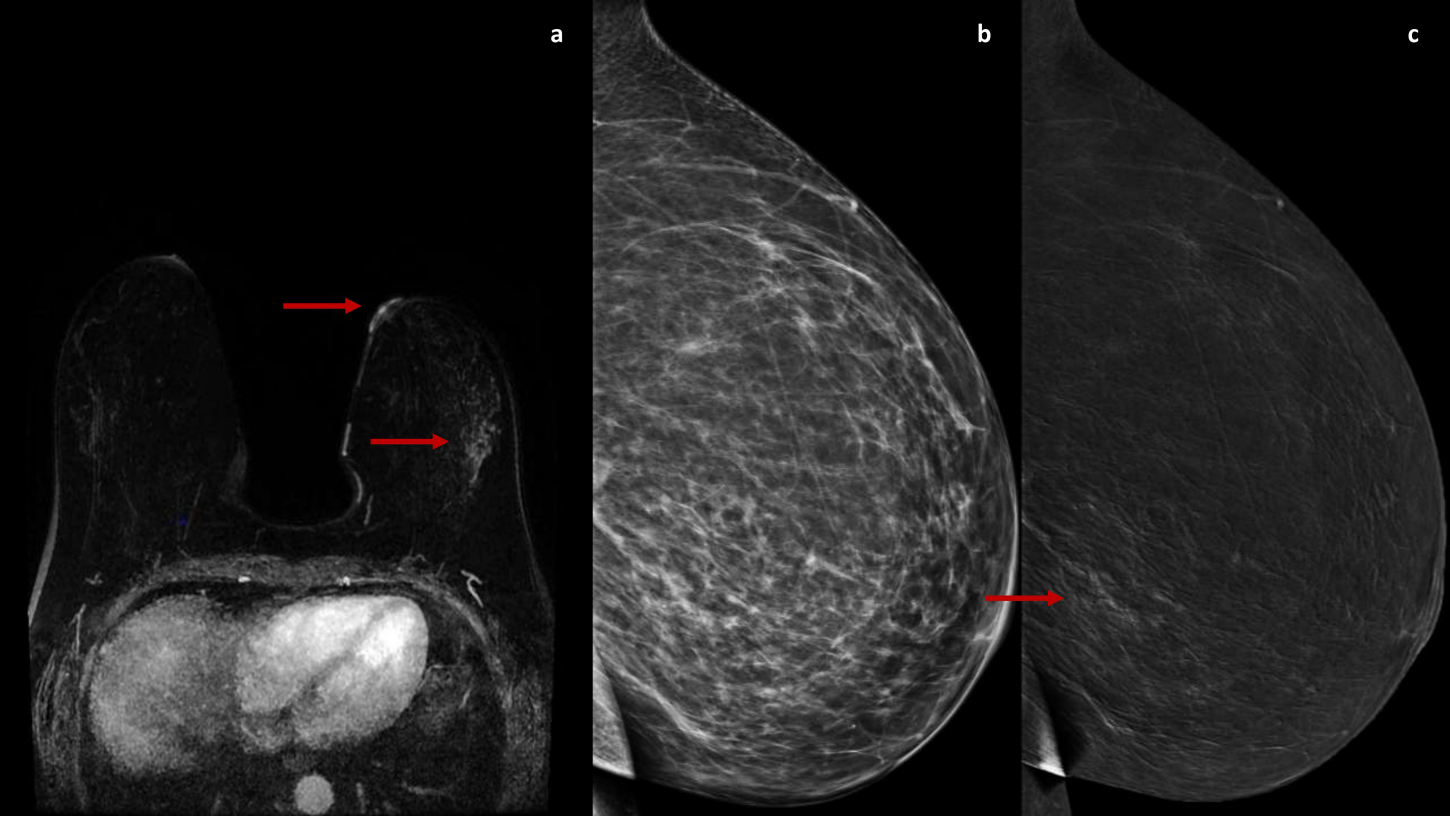
A DCE-MRI subtraction image **(A)** shows an enhancement of the left nipple with a non-mass-like enhancement in the outer quadrants (arrow). The 2D and recombined medio-lateral CEM images **(B, C)** confirm a later inhomogeneous non-mass-like enhancement in the outer quadrants of the left breast (arrow).

**Figure 3:**
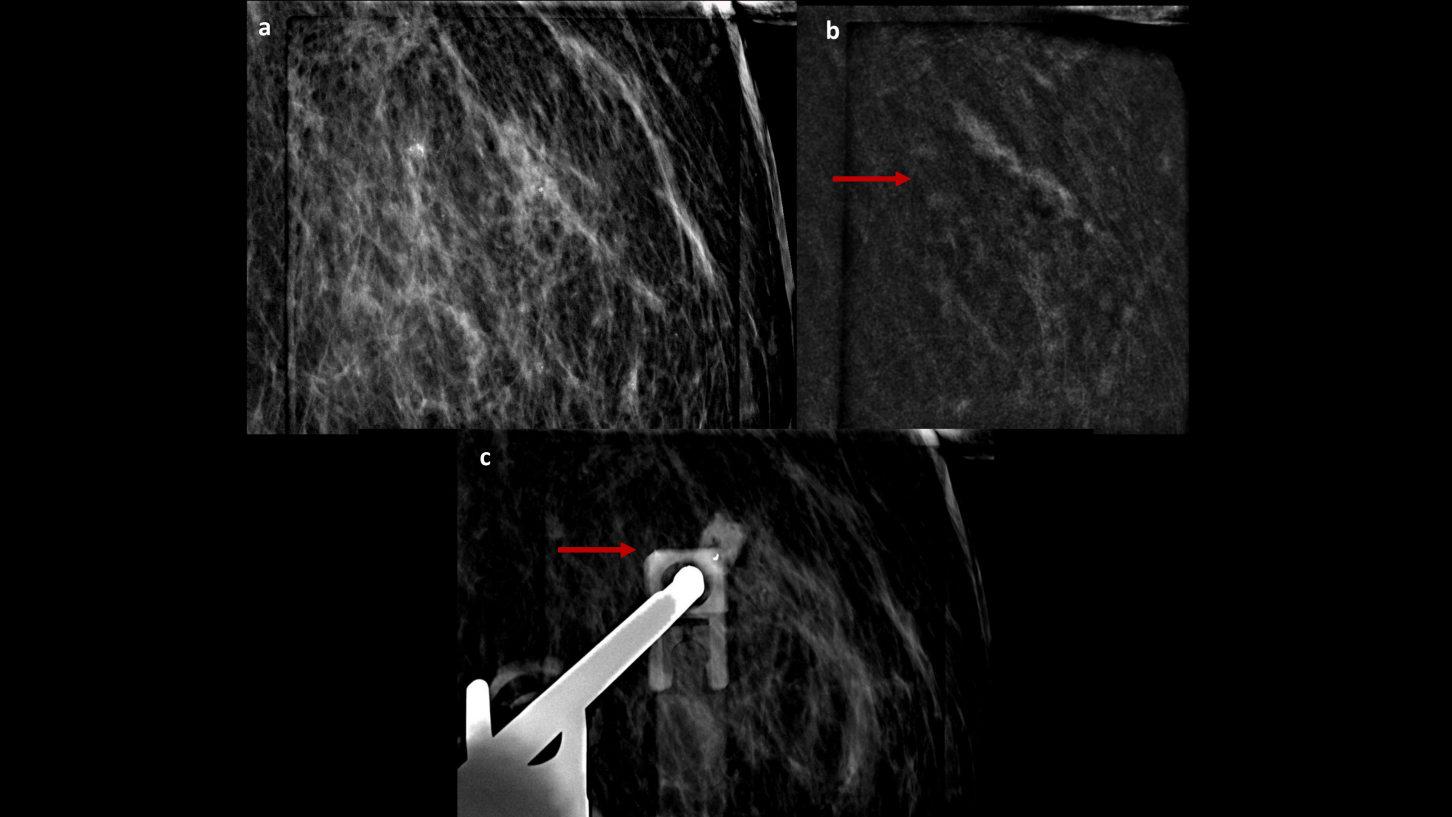
CEM-biopsy: 2D magnification and recombined focus **(A, B)** of the enhancement target in the outer quadrant of the left breast (arrow), where a vacuum-assisted biopsy was performed, followed by the clip placement (**C**, arrow).

The second case (**Figures 2**, **3**) concerns a 57-year-old woman who was referred to our institute due to persistent pain, swelling and eczema of the left nipple, unresponsive to several weeks of antibiotic therapy. The patient was postmenopausal for five years and had not undergone hormone replacement therapy. Clinical examination confirmed marked erythematous eczematous changes in the left NAC without palpable breast masses or axillary adenopathies. DM and US performed at an external facility and did not identify any suspicious findings. However, a NAC punch biopsy performed elsewhere confirmed the PD diagnosis. Due to the discrepancy between the clinical examination and conventional imaging, a breast MRI was performed at the same external center, showing doubtful mild enhancement in the outer quadrants of the left breast. Upon reassessment of the initial DM, some amorphous calcifications were identified in the same region. Consequently, to achieve a morphofunctional correlation between calcifications and the observed enhancement to select a potential biopsy target, a CEM was performed, which confirmed a later inhomogeneous non-mass-like enhancement of approximately 8 cm in the outer quadrants of the left breast, with ipsilateral NAC contrast-enhancement associated. A subsequent CEM-guided biopsy verified a low-grade *in situ* ductal carcinoma (DIN1c) diagnosis. The patient was referred for a left total mastectomy with reconstruction instead of a central resection.

## CEM protocol

CEM was performed using a full-field DM system (Pristina™ mammography system, GE Healthcare, Chalfont St. Giles, United Kingdom). Before breast compression, patients received an automated intravenous single injection of an iodinated contrast agent (Iopromide, 370 mg/ml, 1.5 ml/kg, Ultravist ^®^). Image acquisition started two minutes post-injection, capturing a series of bilateral cranio-caudal (CC) and medio-lateral-oblique (MLO) views starting from the suspicious breast.

## Discussion and conclusions

PD is a rare condition, accounting for 1-3% of all breast malignancies (1). After diagnosis by NAC punch-biopsy, planning appropriate treatment for PD remains challenging for physicians due to the high rate (67-100% of cases) of associated underlying malignancy, often undetectable on conventional imaging techniques (1, 3). Lesions without a discernible mass typically correlate with *in situ* disease confined to the ductal system (DCIS), although they may also suggest the absence of underlying breast malignancy (2). MRI’s high sensitivity in PD detection is well-documented, particularly in assessing the retroareolar region and clinically occult malignancies with negative findings on DM and US, like non-mass enhancement (9). Additionally, MRI is critical for preoperative assessment of the overall extent of disease in patients eligible for breast-conserving therapy (4, 10). However, some of its limitations should be considered. False negatives may arise in low-grade or less aggressive disease forms (6, 7), and false positives can complicate diagnostic specificity (11, 12). Several studies demonstrated that sensitivity for DCIS is variable; some, especially those with a lower pathological grade (G1), can be missed (13–15). Furthermore, cost, availability, and patient-related issues (e.g., claustrophobia, incompatible devices) restrict its routine use.

In this context, CEM could emerge as a valuable alternative or complementary imaging tool in locoregional BC staging and diagnostic problem-solving when other imaging techniques yield inconclusive results. Studies demonstrate a high accuracy of CEM in measuring the main lesion (16–18) and identifying the multifocality and multicentricity of lesions, suggesting how its use in pre-surgical planning may offer significant benefits and surgical plan modification rates similar to MRI (19, 20). A recent Australian prospective investigation compared CEM to MRI for BC staging in 59 women with 68 sites of malignancy, demonstrating statistically equivalent sensitivities of 99% and 97%, respectively (21). CEM can also be evaluated as a tool to characterize additional breast findings that would otherwise be considered indeterminate. Nida et al. assessed the use of CEM as a second look modality to identify correlates of suspicious or indeterminate MRI findings, showing a higher detection fraction of CEM (76/109, 70%) compared to the US (50/109, 46%) (P < 0.001) (22).

To date, few studies have discussed the role of CEM in PD evaluation. Fakhry et al. evaluated the added value of incorporating CEM into the diagnostic workup of PD, demonstrating a higher sensitivity of 97.5% and a similar specificity of 54.2% compared to US-DM and a better performance in the assessment of disease extent, as it was able to detect multifocality, multicentricity, and diffuse abnormalities (8).

The clinical cases we presented support the value of CEM in diagnosing PD. In both cases, CEM led to better preoperative delineation of the disease extension and ultimately changed the surgical strategy. Given its ability to assess lesions’ neo angiogenesis, CEM could be an alternative method to MRI in assessing PD patients, especially when MRI is contraindicated or inconclusive.

Despite its promise, the findings of this presentation must be viewed within the context of limitations. It is based on only two cases from a single center, limiting the generalizability of its conclusions to broader and more diverse populations. However, the absence of quantitative data and statistical comparisons reduces the robustness of the observations.

Future research should address these gaps by conducting larger, multicentric, prospective studies with robust statistical methodologies.

PD of the breast represents a diagnostic and therapeutic challenge, particularly given its frequent association with underlying malignancy, often undetected by conventional imaging techniques. While MRI has historically been the gold standard for detecting clinically occult cancer, aiding in preoperative evaluation, its limitations necessitate the exploration of alternative imaging modalities.

Our cases highlight the potential utility of CEM, demonstrating its effectiveness in preoperative disease delineation and surgical planning. CEM offers several advantages, including comparable sensitivity, greater patient tolerance, and lower costs.

However, these findings must be interpreted cautiously. Future research should focus on multicentric studies with quantitative methodologies to validate CEM’s role and compare it comprehensively with MRI. Addressing these gaps can better establish the full potential of CEM as a diagnostic and planning tool in PD management. Nevertheless, the evidence thus far suggests that CEM could significantly improve PD’s diagnostic workup and treatment planning, mainly when MRI is not feasible.

## Data Availability

The raw data supporting the conclusions of this article will be made available by the authors, without undue reservation.
